# Preclinical perspectives on common iron fortificants and their bioavailability in rat models: a systematic review and meta-analysis

**DOI:** 10.3389/fpubh.2026.1902936

**Published:** 2026-09-03

**Authors:** Jiejia Zhang, Brian L. Lindshield

**Affiliations:** 1 School of Health Sciences, College of Health and Human Sciences, Kansas State University, Manhattan, KS, United States; 2 Department of Clinical Nutrition, Shanghai Eighth People's Hospital, Shanghai, China

**Keywords:** hemoglobin, iron bioavailability, iron fortification, meta-analysis, preclinical study

## Abstract

**Background:**

Numerous preclinical studies on improving iron bioavailability to address iron deficiency anemia have been published; however, no preclinical systematic reviews with pairwise meta-analyses have been conducted. Therefore, it is essential to systematically review, summarize, and evaluate the evidence regarding the effects of iron fortificants in animal studies.

**Methods:**

The initial articles were identified in four scientific databases (PubMed, Web of Science Core Collection, Scopus, and EBSCO) on 10 January 2026. The final included articles were collected after the initial articles were scanned and sorted. The data were extracted for pairwise meta-analysis. Hemoglobin (Hb) was the primary outcome.

**Results:**

A total of 23 studies were included in this systematic review. Data from 13 rat experiments, including 56 comparisons, were analyzed using pairwise meta-analysis. The results of the meta-analysis showed that the overall effects of sodium iron ethylenediaminetetraacetate (NaFeEDTA) and ferrous fumarate on Hb were equivalent to those of ferrous sulfate (FeSO_4_). Ferric pyrophosphate (FePP) and ferric orthophosphate (FePO_4_) were inferior to FeSO_4_.

**Conclusion:**

The meta-analysis results suggest that both NaFeEDTA and ferrous fumarate are effective in improving Hb concentration.

**Systematic review registration:**

https://www.crd.york.ac.uk/PROSPERO/recorddashboard, CRD420261280933.

## 1 Introduction

Anemia is recognized by the World Health Organization (WHO) as a global public health concern and a common comorbidity of many chronic diseases (1). Iron fortification is one of the most cost-effective approaches to address iron deficiency anemia. In theory, the more soluble the iron is, the more it can be absorbed and utilized by the body (2).

The most common iron additives can be divided into three categories based on their water solubility and dilute-acid solubility: (1) water-soluble iron compounds such as ferrous sulfate (FeSO_4_) and sodium iron ethylenediaminetetraacetate (NaFeEDTA) (3); (2) water-insoluble but dilute-acid-soluble iron compounds such as ferrous fumarate (FeC_4_H_2_O) (3); and (3) water-insoluble and dilute-acid-insoluble iron compounds such as ferric orthophosphate (FePO_4_) and ferric pyrophosphate (FePP) (4). C_4_H_2_FeO_4_ is often used as an optimal positive control or gold standard because it can be dissolved in water to provide relatively high levels of bioavailable iron; however, it has substandard organoleptic qualities (5). NaFeEDTA is a chelate that has a special structure: an octahedral complex with ferric iron at its center. It is formed when ethylenediaminetetraacetate (EDTA) chelates ferric iron through six coordinate covalent bonds. It has high iron bioavailability without undesirable colors or flavors (5, 6). Ferrous fumarate is a ferrous complex. It is moderately soluble in water, while its sensory characteristics can also be altered. Ferrous fumarate dissolves in the acidic environment of the stomach and can then be absorbed in the more alkaline environment of the duodenum. Thus, it has good solubility and bioavailability. Moreover, it was found that encapsulation technology could enhance the iron absorption of ferrous fumarate in the gastrointestinal tract (7–9). FePO_4_ and FePP do not affect the sensory properties of the food matrix; however, they have low solubility and bioavailability (4). Ideally, iron fortificants should possess high iron bioavailability without altering sensory properties.

The efficacy of iron fortificants in humans depends on multiple factors such as type, concentration, food matrix, enhancers (e.g., citric acid, ascorbic acid, etc.), inhibitors (e.g., polyphenols, tannins, phytic acid, etc.), food processing approaches (e.g., precooking, extrusion, etc.), and physiological and pathological conditions (10). In this systematic review, *in vivo* animal studies were examined. The population (P) consisted of rats. The interventions (I) included four common iron fortificants, namely FePP, FePO_4_, ferrous fumarate, and NaFeEDTA. The comparison (C) was made against the positive control, FeSO_4_. The primary outcome (O) was hemoglobin (Hb) concentration. Hb was selected as the primary outcome for evaluating iron bioavailability because it is a well-established indicator of iron deficiency anemia. Following intestinal absorption, most dietary iron is transported to the bone marrow for Hb synthesis, with approximately 70% of the body’s iron being incorporated into Hb for oxygen transport. Therefore, monitoring Hb concentration in depletion–repletion rat models is one of the most reliable approaches for assessing the bioavailability of iron fortificants (11, 12). The secondary outcomes included, but were not limited to, plasma/serum iron, hematocrit, transferrin saturation, total iron-binding capacity, iron solubility, hepatic iron, and bone marrow iron, which were the indicators in the assessment of early negative iron storage status and iron depletion status (13).

To the best of our knowledge, no preclinical systematic reviews with pairwise meta-analyses on iron bioavailability have been published. The PICO question addressed in this systematic review was as follows: In rat models, does iron fortification, compared to a positive control, prevent or improve iron deficiency and/or anemia? The findings of relevant preclinical studies on four common iron fortificants were identified, evaluated, and summarized. The process is schematically shown in Supplementary material 1.

## 2 Materials and methods

### 2.1 Eligibility criteria

#### 2.1.1 Inclusion criteria

The studies included in this analysis had to involve Sprague–Dawley rats. At least one targeted iron fortificant with an identified iron type and quantity had to be provided in a food matrix. At least one primary or secondary outcome had to be reported. The manuscript had to be a peer-reviewed journal article written in English. Studies with a completely randomized design were included in the meta-analysis. For the meta-analysis, studies that included FeSO_4_ as a positive control were included.

#### 2.1.2 Exclusion criteria

Studies using pregnant rats or other animal species such as piglets, chickens, or mice, (including Wistar rats) were excluded. Studies focused on iron supplementation and those involving special food (e.g., testing foods that were naturally high in iron, etc.) were not included. Human research, clinical trials, observational studies (e.g., cohort and case–control studies), case reports, reviews, protocols, theses, dissertations, book sections, abstracts, proceedings, and non-primary research were excluded. Cross-over designs were also excluded. For the meta-analysis, studies using placebo or blank controls were not included.

### 2.2 Search strategies

We carried out the search strategy across four scientific databases (PubMed, Web of Science Core Collection, Scopus, and EBSCO Agricola) on 10 January 2026. To broaden the search for additional articles, snowball and citation search methods were employed in other academic search engines (e.g., Google Scholar). The search items and keywords were developed in accordance with each database’s user guide and classified into five categories, as detailed in Supplementary material 2. All relevant studies were identified in the “all fields” or “topic” fields by using advanced search builder and Boolean operators. The filters were applied to specify publication types for peer-reviewed articles published in English.

### 2.3 Data management, data extraction, and quality assessments

The initial articles from four databases were imported into an online data manager, Zotero. After automatic duplicate/triplicate/quadruplicate merging, the articles screened by titles and abstracts were manually scanned. The remaining articles were thoroughly reviewed and further sorted to obtain the final included articles (14). The scanning and sorting processes were performed by two researchers. The data and characteristics of the included studies were extracted by a single reviewer using Microsoft Excel. The extracted information included authors, years of publication, iron types, iron concentration, enhancing agents, animal types (species and gender), intervention duration, sample sizes, model types (prophylactic–preventive method or depletion-repletion method), and outcomes.

The quality assessments of included studies were conducted according to the Collaborative Approach to Meta-Analysis and Review of Animal Data from Experimental Studies (CAMARADES) checklist, SYstematic Review Center for Laboratory animal Experimentation (SYRCLE) guide, and PRISMA checklist. Any disagreements between the two reviewers were resolved through discussion and consensus. If consensus could not be reached, a third senior reviewer adjudicated. The quality assessments consisted of 10 items, each evaluated as Yes, No, or Unclear in accordance with the modified “Risk of Bias” tools described in the Cochrane Handbook (15–18): (1) Allocation sequence generation, (2) Baseline characteristics, (3) Allocation concealment, (4) Random housing, (5) Intervention blinding, (6) Random outcome assessment, (7) Outcome blinding, (8) Incomplete outcome data, (9) Selective outcome reporting, and (10) Other sources of bias (15–18).

### 2.4 Statistical analysis

The analyses were performed using Review Manager (version 5.4, Cochrane’s bespoke software) and Stata (version 18.0, USA), with significance set at *p* < 0.05. In the meta-analysis, the mean difference (MD) and 95% confidence interval (CI) were used to assess the overall effect of the continuous outcome. When only the standard error of the mean (SEM) was reported, standard deviation (SD) could be calculated using the formula SD = SEM × √n, where n represents the number of animals (19). Heterogeneity among studies was assessed using the I^2^, χ^2^, and τ^2^ tests (20, 21). A random-effects model was used for pooling data. Publication bias was assessed using Begg’s test and Egger’s test and was displayed in a funnel plot. A symmetrical funnel plot suggested a lower likelihood of publication bias and vice versa (16, 20–26). A subgroup analysis was conducted to assess the pooled effect within individual subgroups. The t-test was used to compare the means of groups. If one control group served multiple experimental groups, each control–experimental pairing was treated as a separate comparison. The certainty of evidence was assessed using the Grading of Recommendations Assessment, Development and Evaluation (GRADE) framework and the GRADEpro GDT online tool^1^ (16, 20–26).

## 3 Results

### 3.1 Description of search results

**Figure 1:**
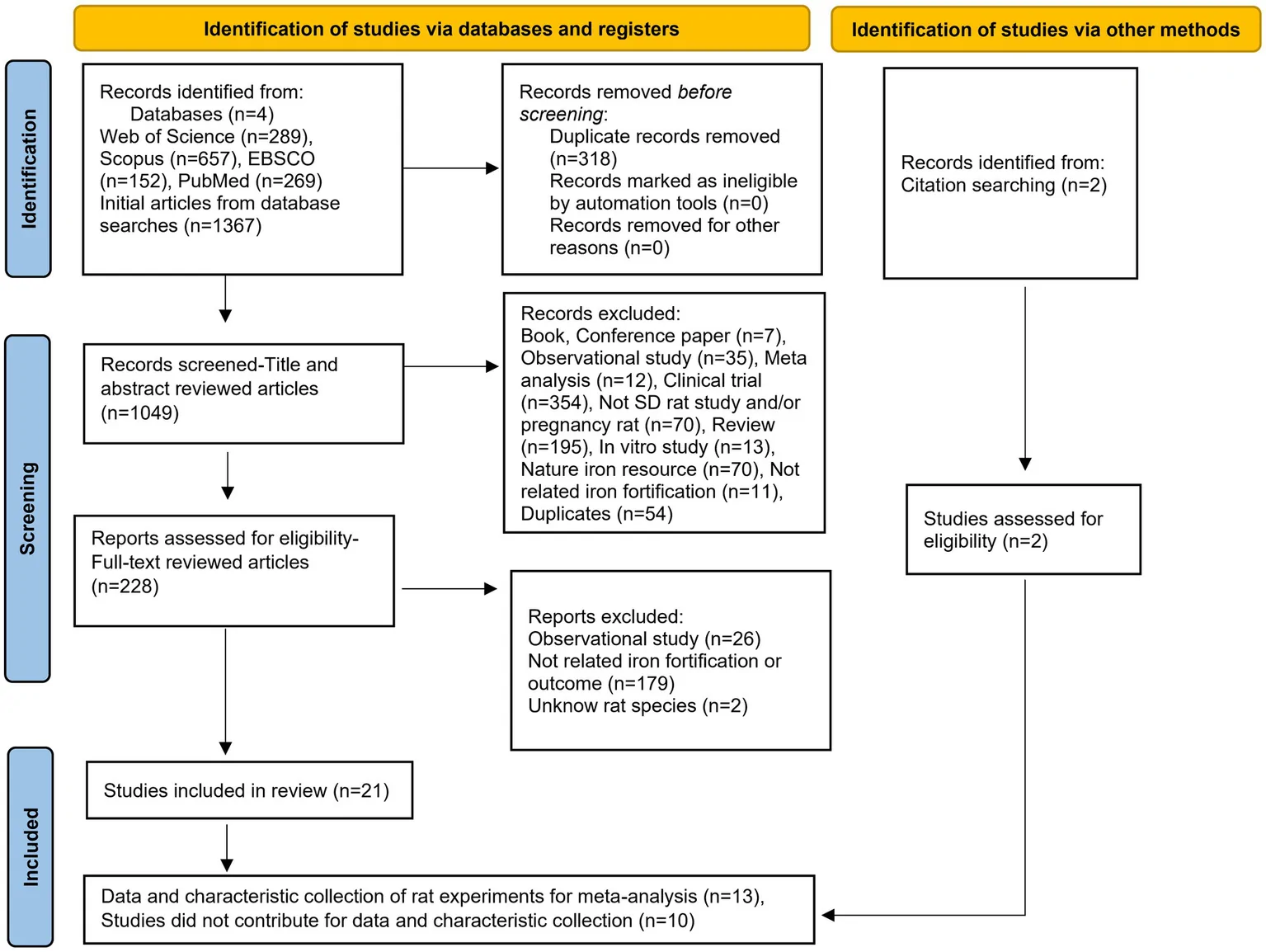
Flow diagram of scanning and sorting studies.

The comprehensive search yielded 1,367 initial articles. Two additional articles were added. After merging 318 iterations and screening 1,051 titles and abstracts, 821 articles were excluded. The full texts of the remaining 230 articles were reviewed, and 207 studies were further excluded. The number of final included articles was 23. The sorting and screening process is shown in Figure 1. In all the included studies, 13 rat experiments, comprising 56 comparisons and involving 924 rats, were analyzed by pairwise meta-analysis and contributed to the summary of data and characteristics. The remaining animal studies could not be included in the meta-analysis due to the lack of outcome data at baseline or endline or because FeSO_4_ was not used as a positive control. There were insufficient experiments involving other species for pairwise meta-analysis.

### 3.2 Characteristics and quality assessments of included studies

The characteristics of 13 studies with 56 comparisons were summarized in Tables 1–4. Among these, 10 studies (76.9%) used depletion (7–35 days)-repletion (7–28 days) models and 3 studies (23.1%) used prophylactic-preventative (21–60 days) models. The food matrix in 10 studies was grain-based diets (*n* = 10, 76.9%). One study (*n* = 1, 7.7%) investigated both the grain-based diet matrix and the yogurt dietary matrix. The other matrices (*n* = 2, 15.4%) were cheese and fruit juice. The predominant particle size of iron fortificants was regular size (20–30 μm, *n* = 7, 53.9%). One study (7.7%) examined both the regular-sized iron and the micronized iron (0.5–2.5 μm). Three studies focused on micronized iron (0.5–6 μm, 23.1%), while two studies investigated nanoparticles (10–64 nm, 15.4%) iron. The iron fortification level across the 13 studies ranged from 10 to 59 mg Fe/kg of food.

**Table 1:** Characteristics of FePO_4_ rat studies in the meta-analysis.

Study	Species	Sample size Fe/control	Intervention model	Intervention duration (days)	Food matrix	Iron type	Particle size	Iron concentration (Fe mg/kg food)	Control	Primary outcome: Hb gain (g/dL)	Secondary outcomes	Comparison no.
Rohner 2007 (41)	Male S-D rats	9/10	Depletion-repletion	Depletion: 24 Repletion: 15	AIN-93G	FePO_4_	Nanoparticle (64 nm)	10	FeSO_4_	No significant difference	Plasma TBARS: No significant difference	1
		10/10			AIN-93G	FePO_4_	Nanoparticle (31 nm)	10	FeSO_4_	FePO_4_ < FeSO_4_		2
		10/10			AIN-93G	FePO_4_	Nanoparticle (11 nm)	10	FeSO_4_	No significant difference		3
		10/10			AIN-93G	FePO_4_	Nanoparticle (64 nm)	20	FeSO_4_	FePO_4_ < FeSO_4_		4
		10/10			AIN-93G	FePO_4_	Nanoparticle (31 nm)	20	FeSO_4_	FePO_4_ < FeSO_4_		5
		10/10			AIN-93G	FePO_4_	Nanoparticle (11 nm)	20	FeSO_4_	FePO_4_ < FeSO_4_		6
Dickmann 2016 (50)	Male S-D rats	10/4	Depletion-repletion	Depletion: 15 Repletion: 21	AIN-76A	FePO_4_ (Source No.1)	Regular	6	FeSO_4_	No significant difference	Not reported	7
		10/4			AIN-76A	FePO_4_ (Source No.2)	Regular	6	FeSO_4_	No significant difference		8
		10/4			AIN-76A	FePO_4_ (Source No.3)	Regular	6	FeSO_4_	No significant difference		9
		10/4			AIN-76A	FePO_4_ (Source No.4)	Regular	6	FeSO_4_	No significant difference		10
		10/4			AIN-76A	FePO_4_ (Source No.5)	Regular	6	FeSO_4_	No significant difference		11
		10/10			AIN-76A	FePO_4_ (Source No.1)	Regular	12	FeSO_4_	No significant difference		12
		10/10			AIN-76A	FePO_4_ (Source No.2)	Regular	12	FeSO_4_	FePO_4_ < FeSO_4_		13
		10/10			AIN-76A	FePO_4_ (Source No.3)	Regular	12	FeSO_4_	FePO_4_ < FeSO_4_		14
		10/10			AIN-76A	FePO_4_ (Source No.4)	Regular	12	FeSO_4_	FePO_4_ < FeSO_4_		15
		10/10			AIN-76A	FePO_4_ (Source No.5)	Regular	12	FeSO_4_	FePO_4_ < FeSO_4_		16
		10/5			AIN-76A	FePO_4_ (Source No.1)	Regular	24	FeSO_4_	No significant difference		17
		10/5			AIN-76A	FePO_4_ (Source No.2)	Regular	24	FeSO_4_	No significant difference		18
		10/5			AIN-76A	FePO_4_ (Source No.3)	Regular	24	FeSO_4_	FePO_4_ < FeSO_4_		19
		10/5			AIN-76A	FePO_4_ (Source No.4)	Regular	24	FeSO_4_	No significant difference		20
		10/5			AIN-76A	FePO_4_ (Source No.5)	Regular	24	FeSO_4_	FePO_4_ < FeSO_4_		21
Whittaker 1990 (39)	Male S-D rats	10	Depletion-repletion	Depletion: 7 Repletion: 10	AIN-76A	FePO_4_	Regular	35	FeSO_4_	FePO_4_ < FeSO_4_	Hepatic iron (μg): FePO_4_ < FeSO_4_	22
		10			AIN-76A	FePO_4_ + ascorbic acid	Regular	35	FeSO_4_ + ascorbic acid	FePO_4_ < FeSO_4_	Hepatic iron (μg): FePO_4_ < FeSO_4_	23
Lysionek 2002a (51)	Female S-D rats	10	Prophylactic-preventative	Intervention: 23	Petit-Suisse Cheese	FePO_4_	Micronized (6 μm)	30	FeSO_4_	FePO_4_ < FeSO_4_	Hepatic iron (mg): No significant difference	24

“>” indicates a significantly higher effect; “<” indicates a significantly lower effect. AIN-76A, American Institute of Nutrition 1976 diet; AIN-93G, American Institute of Nutrition 1993 growth diet; FePO4, ferric orthophosphate; FeSO4, ferrous sulfate; Hb, hemoglobin; S-D, Sprague–Dawley; TBARS, thiobarbituric acid-reactive substances.

**Table 2:** Characteristics of FePP rat studies in the meta-analysis.

Study	Species	Sample size	Intervention model	Intervention duration (days)	Food matrix	Iron type	Particle size	Iron concentration (Fe mg/kg food)	Control	Primary outcome: Hb gain (g/dL)	Secondary outcomes	Comparison no.
Srinivasu 2015 (52)	Male S-D rats	8	Depletion-repletion	Depletion: 30 Repletion: 15	AIN-93G	FePP	Nanoparticle (10–30 nm)	10	FeSO_4_	No significant difference	Not reported	25
		8				FePP	Nanoparticle (10–30 nm)	20	FeSO_4_	No significant difference		26
		8				FePP	Nanoparticle (10–30 nm)	30	FeSO_4_	FePP < FeSO_4_		27
Haro- Vicente 2008 (53)	Male S-D rats	6	Depletion-repletion	Depletion: 28 Repletion: 21	Fruit juice	FePP	Micronized (0.5 μm)	46	FeSO_4_	No significant difference	Hepatic iron (mg/g): FePP <FeSO_4._	28
		6			Fruit juice	FePP+ascorbic acid	Micronized (0.5 μm)	46	FeSO_4_ + ascorbic acid	No significant difference	Hepatic iron (mg/g): No significant difference.	29
Sakaguchi 2004 (54)	Male S-D rats	6	Depletion-repletion	Depletion: 35 Repletion: 28	Corn starch	FePP	Micronized (0.5 μm)	35	FeSO_4_	No significant difference	Not reported	30
		6				FePP	Micronized (5.2 μm)	35	FeSO_4_	FePP <FeSO_4_		31
		6		Depletion: 35 Repletion: 14		FePP	Micronized (0.5 μm)	12	FeSO_4_	No significant difference		32
		6				FePP	Micronized (5.2 μm)	12	FeSO_4_	FePP <FeSO_4_		33
		6		Depletion: 35 Repletion: 14		FePP	Micronized (0.5 μm)	24	FeSO_4_	No significant difference		34
		6				FePP	Micronized (5.2 μm)	24	FeSO_4_	FePP <FeSO_4_		35
Wegmüller 2004 (55)	Male S-D rats	8	Depletion-repletion	Depletion: 24 Repletion: 14	AIN-93G	FePP	Regular	20	FeSO_4_	FePP <FeSO_4_	Not reported	36
		8			AIN-93G	FePP	Micronized (2.5 μm)	20	FeSO_4_	FePP <FeSO_4_		37
		8			AIN-93G	FePP(Encapsulated)	Micronized (2.5 μm)	20	FeSO_4_	FePP <FeSO_4_		38
		8			AIN-93G	FePP	Small micronized (0.5 μm)	20	FeSO_4_	No significant difference		39
		8			AIN-93G	FePP	Regular	10	FeSO_4_	FePP <FeSO_4_		40
		8			AIN-94G	FePP	Micronized (2.5 μm)	10	FeSO_4_	FePP <FeSO_4_		41
		8			AIN-95G	FePP(Encapsulated)	Micronized (2.5 μm)	10	FeSO_4_	FePP <FeSO_4_		42
		8			AIN-96G	FePP	Small micronized (0.5 μm)	10	FeSO_4_	No significant difference		43
Salgueiro 2009 (56)	Male S-D rats	10	Prophylactic-preventative	Intervention:21	AIN-93G	FePP	Regular	16.25	FeSO_4_	No significant difference	Not reported	44
		10			Yogurt	FePP	Regular	15.4	FeSO_4_	FePP >FeSO_4_		45

“>” indicates a significantly higher effect; “<” indicates a significantly lower effect. AIN-93G, AIN-94G, AIN-95G, and AIN-96G, American Institute of Nutrition growth diets; FePO4, ferric orthophosphate; FePP, ferric pyrophosphate; FeSO4, ferrous sulfate; Hb, hemoglobin; S-D, Sprague–Dawley.

**Table 3:** Characteristics of NaFeEDTA rat studies in the meta-analysis.

Study	Species	Sample size	Intervention model	Intervention duration (days)	Food matrix	Iron type	Particle size	Iron concentration (Fe mg/kg food)	Control	Primary outcome-final Hb value (g/L)	Secondary outcomes	Comparison no.
Zhu 2006 (6)	Male S-D rats	6	Depletion-repletion	Depletion:7 Repletion: 12	AIN-93G	NaFeEDTA	Regular	35	FeSO_4_	No significant difference	Hepatic iron (μg/g): No significant difference Bone iron (μg/g): No significant difference.	46
Malika 2022 (57)	S-D rats	3	Prophylactic-preventative	Intervention:60	Wheat flour	NaFeEDTA	Regular	15	FeSO_4_	No significant difference	Total iron binding capacity (μg/dL): No significant difference.	47
		3				NaFeEDTA	Regular	25	FeSO_4_	No significant difference	Total iron binding capacity (μg/dL): No significant difference.	48
		3				NaFeEDTA	Regular	35	FeSO_4_	No significant difference	Total iron binding capacity (μg/dL): No significant difference.	49
		3				NaFeEDTA	Regular	40	FeSO_4_	No significant difference	Total iron binding capacity (μg/dL): No significant difference.	50
Ologunde 1994 (58)	Male S-D rats	10	Depletion-repletion	Depletion: 7 Repletion: 7	AIN-76A	NaFeEDTA	Regular	35	FeSO_4_	No significant difference	Hematocrit (%): No significant difference Total iron binding capacity (μg/dL): No significant difference; Serum iron (μg/dL): No significant difference; Hepatic iron (μg): NaFeEDTA> FeSO_4_.	51

Whittaker 1990 (39)	Male S-D rats	10	Depletion-repletion	Depletion: 7 Repletion: 10	AIN-76A	NaFeEDTA	Regular	30	FeSO_4_	NaFeEDTA >FeSO_4_	Hepatic iron (μg): No significant difference	52
		10			Bread	NaFeEDTA	Regular	30	FeSO_4_	NaFeEDTA >FeSO_4_	Hepatic iron (μg): No significant difference.	53

“>” indicates a significantly higher effect; “<” indicates a significantly lower effect. AIN-76A, American Institute of Nutrition 1976 diet; AIN-93G, American Institute of Nutrition 1993 growth diet; FeSO4, ferrous sulfate; Hb, hemoglobin; NaFeEDTA, sodium iron ethylenediaminetetraacetate; S-D, Sprague–Dawley.

**Table 4:** Characteristics of ferrous fumarate rat studies in the meta-analysis.

Study	Species	Sample size	Intervention model	Intervention duration (days)	Food matrix	Iron type	Particle size	Iron concentration (Fe mg/kg food)	Control	Primary outcome-final Hb value (g/L)	Secondary outcomes	Comparison no.
Hernández 2003 (59)	Male and female S-D rats	6	Depletion-repletion	Depletion: 10 Repletion: 14	Corn flour	Ferrous fumarate	Regular	56	FeSO_4_	No significant difference	Not reported	54
		6			Wheat flour	Ferrous fumarate	Regular	59	FeSO_4_	No significant difference		55
Ologunde 1994 (58)	Male S-D rats	10	Depletion-repletion	Depletion: 7 Repletion: 7	AIN-76A	Ferrous fumarate	Regular	35	FeSO_4_	No significant difference	Hematocrit (%): No significant difference Total iron binding capacity (μg/dL): Fumarate < FeSO_4_; Serum iron (μg/dL): No significant difference; Hepatic iron (μg): Fumarate> FeSO_4_	56

“>” indicates a significantly higher effect; “<” indicates a significantly lower effect. AIN-76A, American Institute of Nutrition 1976 diet; FeSO4, ferrous sulfate; Hb, hemoglobin; S-D, Sprague–Dawley.

As shown in the quality assessments in Table 5, none of the studies reported the methods used for sequence generation. Nine studies had similar baseline characteristics, whereas four studies did not clearly describe the baseline levels. No studies reported whether the allocation was concealed. Rats were randomly housed in eight studies. Five studies did not report whether housing was randomized. In 13 studies, the caregivers or investigators were not blinded. No studies reported information on randomized outcome assessment. One study stated that the outcome assessors were unaware of the group assignments. The remaining studies did not mention whether the outcome assessor was blinded. One study had incomplete outcome data but did not clarify how it was addressed. For the other studies, the handling of incomplete outcome data was unclear. No study had other potential sources of bias. The systematic review was assessed in accordance with the PRISMA Checklist and met all PRISMA Checklist requirements except for the sensitivity analysis items (13f, 20d). A sensitivity analysis was not performed because of insufficient data.

**Table 5:** Quality assessments of included studies.

ID	Authors, year of publication	1	2	3	4	5	6	7	8	9	10
1	Rohner 2007 (41)	N	Y	N	Y	N	N	Y	N	N	N
2	Dickmann 2016 (50)	N	N	N	Y	N	N	N	U	N	N
3	Whittaker 1990 (39)	N	Y	N	Y	N	N	N	U	N	N
4	Lysionek 2002a (51)	N	N	N	N	N	N	N	U	N	N
5	Srinivasu 2015 (52)	N	N	N	Y	N	N	N	U	N	N
6	Vicente 2008 (53)	N	Y	N	N	N	N	N	U	N	N
7	Sakaguchi 2004 (54)	N	Y	N	Y	N	N	N	U	N	N
8	Wegmüller 2004 (55)	N	Y	N	Y	N	N	N	U	N	N
9	Salgueiro 2009 (56)	N	Y	N	Y	N	N	N	U	N	N
10	Zhu 2006 (6)	N	Y	N	Y	N	N	N	U	N	N
11	Malika 2022 (57)	N	N	N	N	N	N	N	U	N	N
12	Ologunde 1994 (58)	N	Y	N	N	N	N	N	U	N	N
13	Hernández 2003 (59)	N	Y	N	N	N	N	N	U	N	N

“1” represents “sequence generation.” “2” represents “baseline characteristics.” “3” represents “allocation concealment.” “4” represents “random housing.” “5” represents “intervention blinding.” “6” represents “random outcome assessment.” “7” represents “outcome blinding.” “8” represents “incomplete outcome data.” “9” represents “selective outcome reporting.” “10” represents “other sources of bias.” “Y” represents “Yes.” “N” represents “No.” “U” represents “Unclear.” ID, identifier.

### 3.3 Pairwise meta-analysis results

#### 3.3.1 FePO_4_ fortification vs. FeSO_4_ fortification

**Figure 2:**
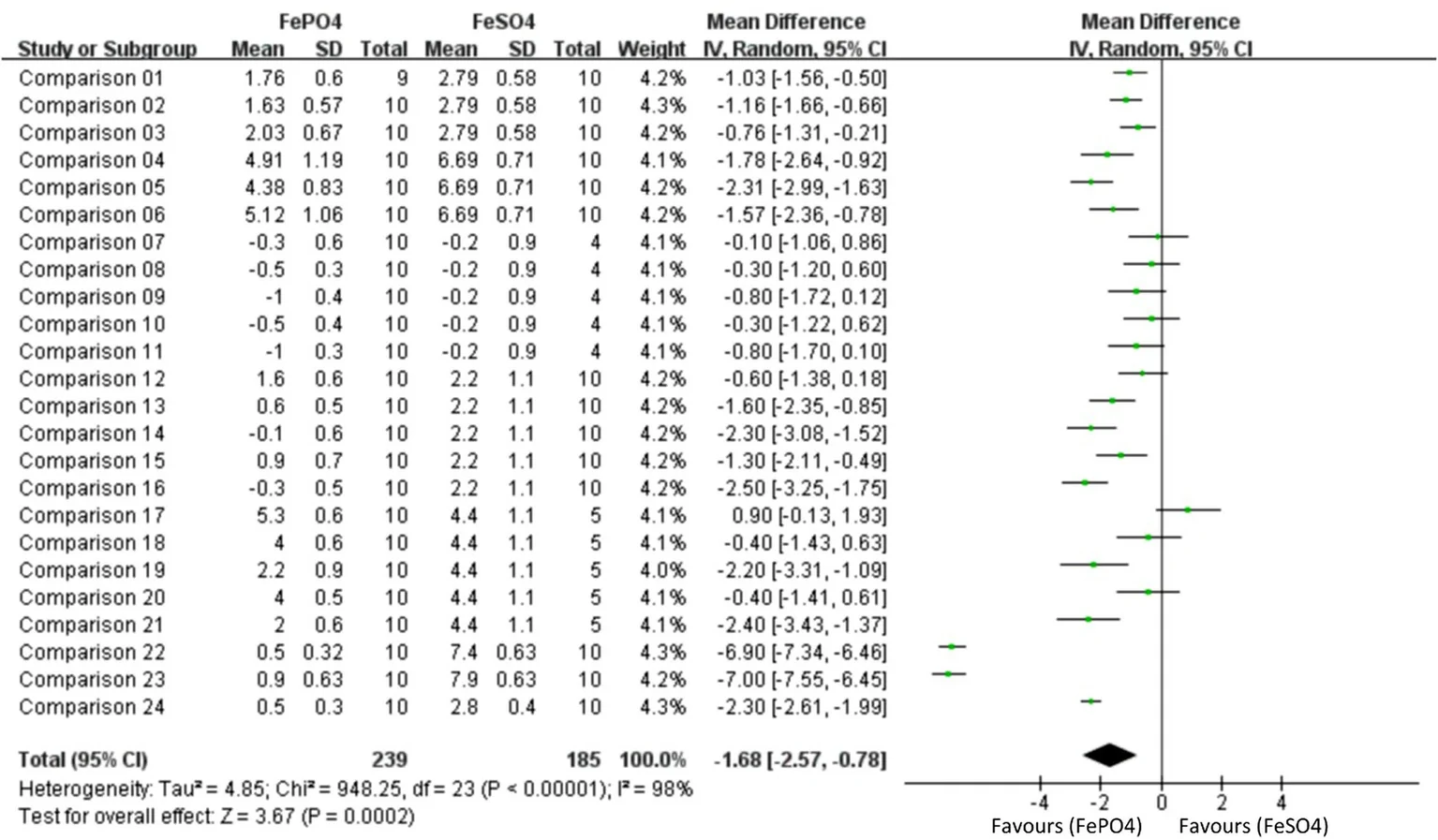
Ferric orthophosphate (FePO_4_) vs. ferrous sulfate (FeSO_4_) forest plot.

**Figure 3:**
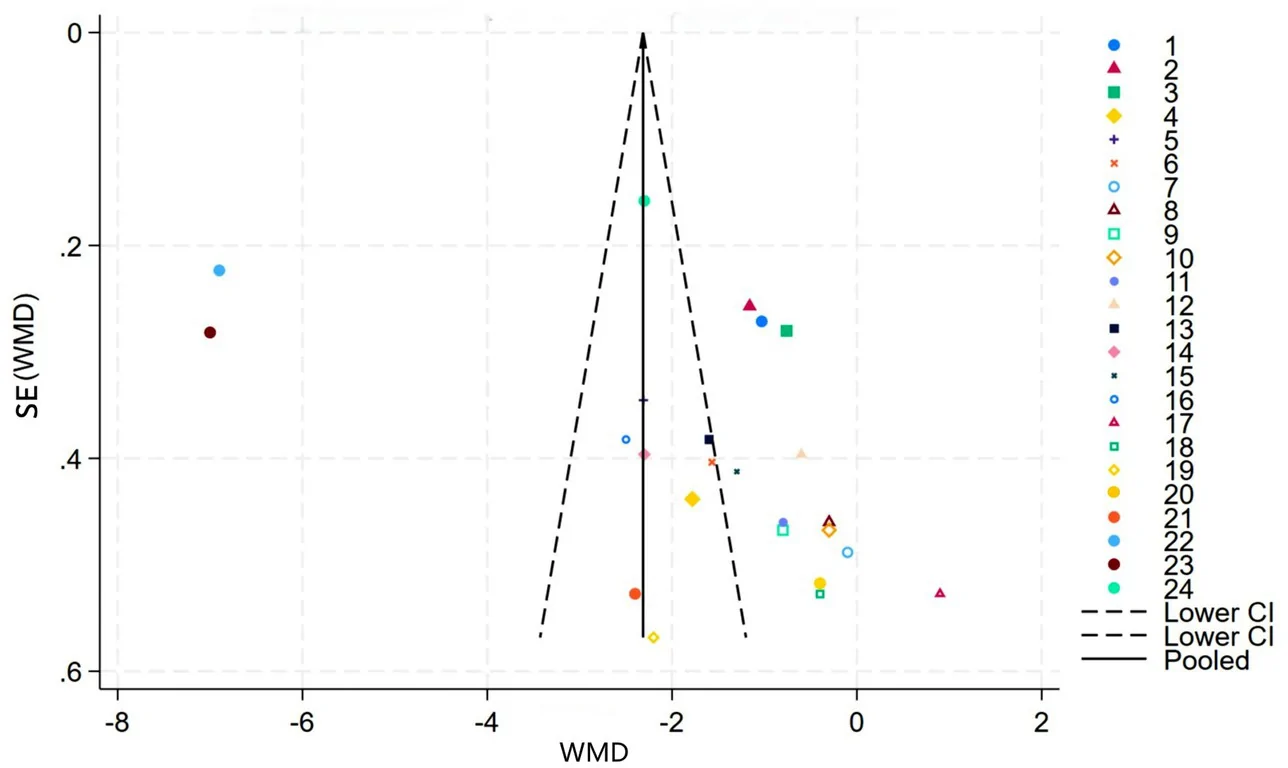
Ferric orthophosphate (FePO_4_) vs. ferrous sulfate (FeSO_4_) funnel plot.

**Figure 4:**
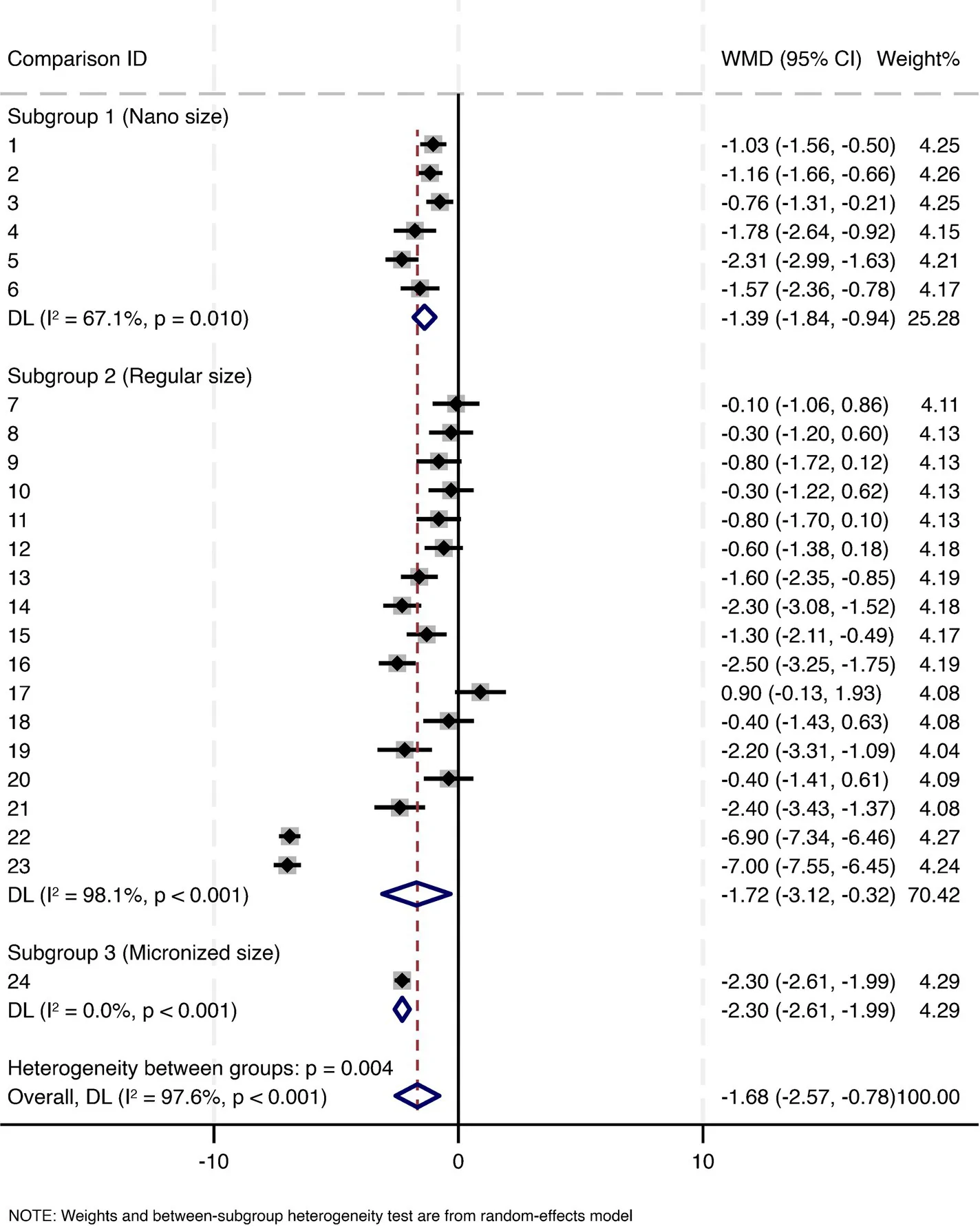
Ferric orthophosphate (FePO_4_) vs. ferrous sulfate (FeSO_4_) subgroup (particle size) funnel plot.

Four studies including 24 comparisons and 424 rats assessed Hb gain (g/dL). Compared to the rats receiving FeSO_4_ fortification, the rats receiving FePO_4_ fortification had lower Hb gain (MD = −1.68; 95% CI, −2.57 to −0.78, *p* < 0.001) with high heterogeneity (I^2^ = 98%; Figure 2). The significant publication bias was indicated by Begg’s test (*p* = 0.001) and Egger’s test (*p* < 0.001) and was displayed in an asymmetrical funnel plot (Figure 3). A significant subgroup effect was detected among nanoparticle FePO_4_, micronized FePO_4_, and regular FePO_4_ (*p* = 0.004; Figure 4).

#### 3.3.2 FePP fortification vs. FeSO_4_ fortification

**Figure 5:**
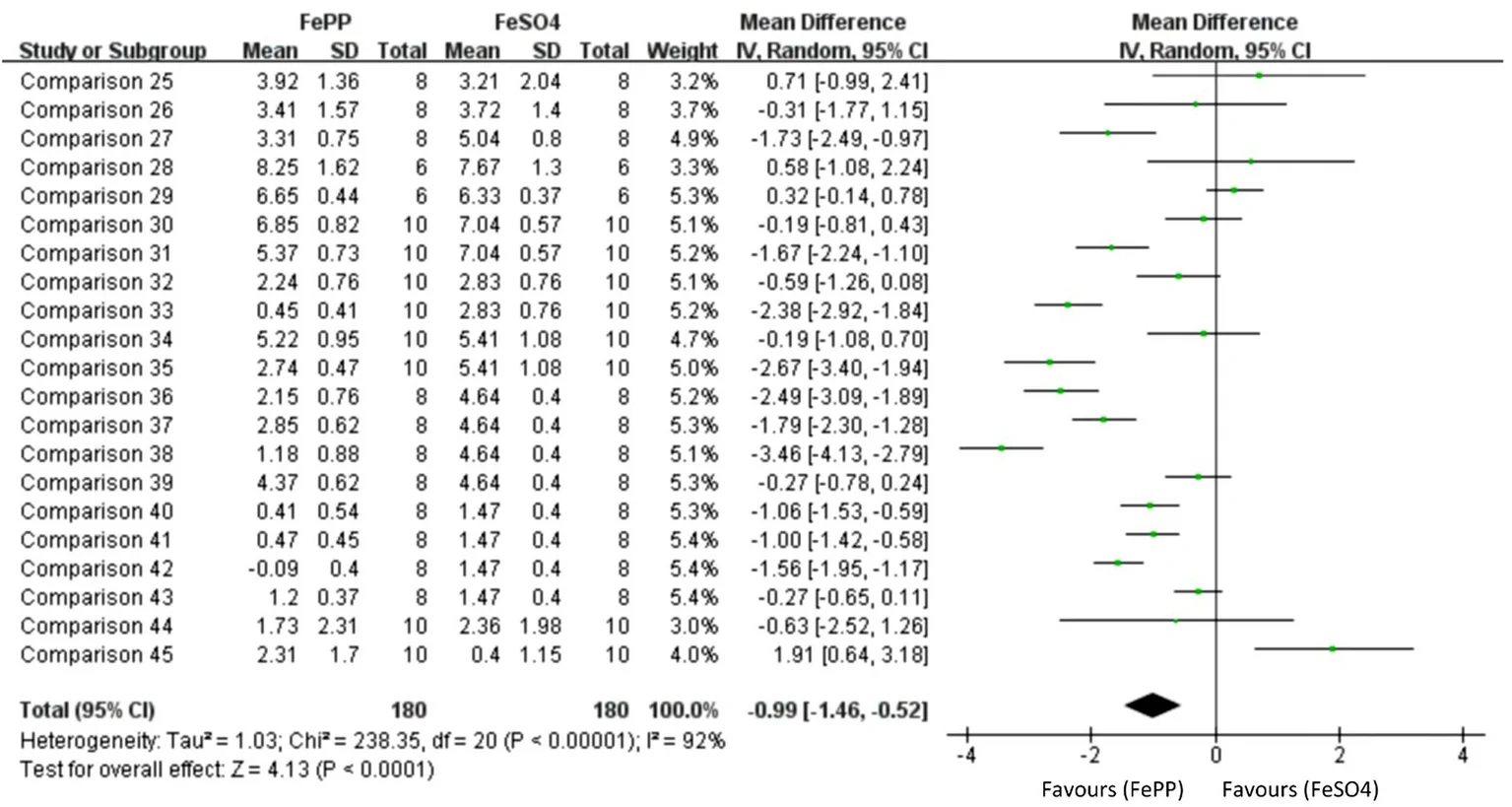
Ferric pyrophosphate (FePP) vs. ferrous sulfate (FeSO_4_) forest plot.

**Figure 6:**
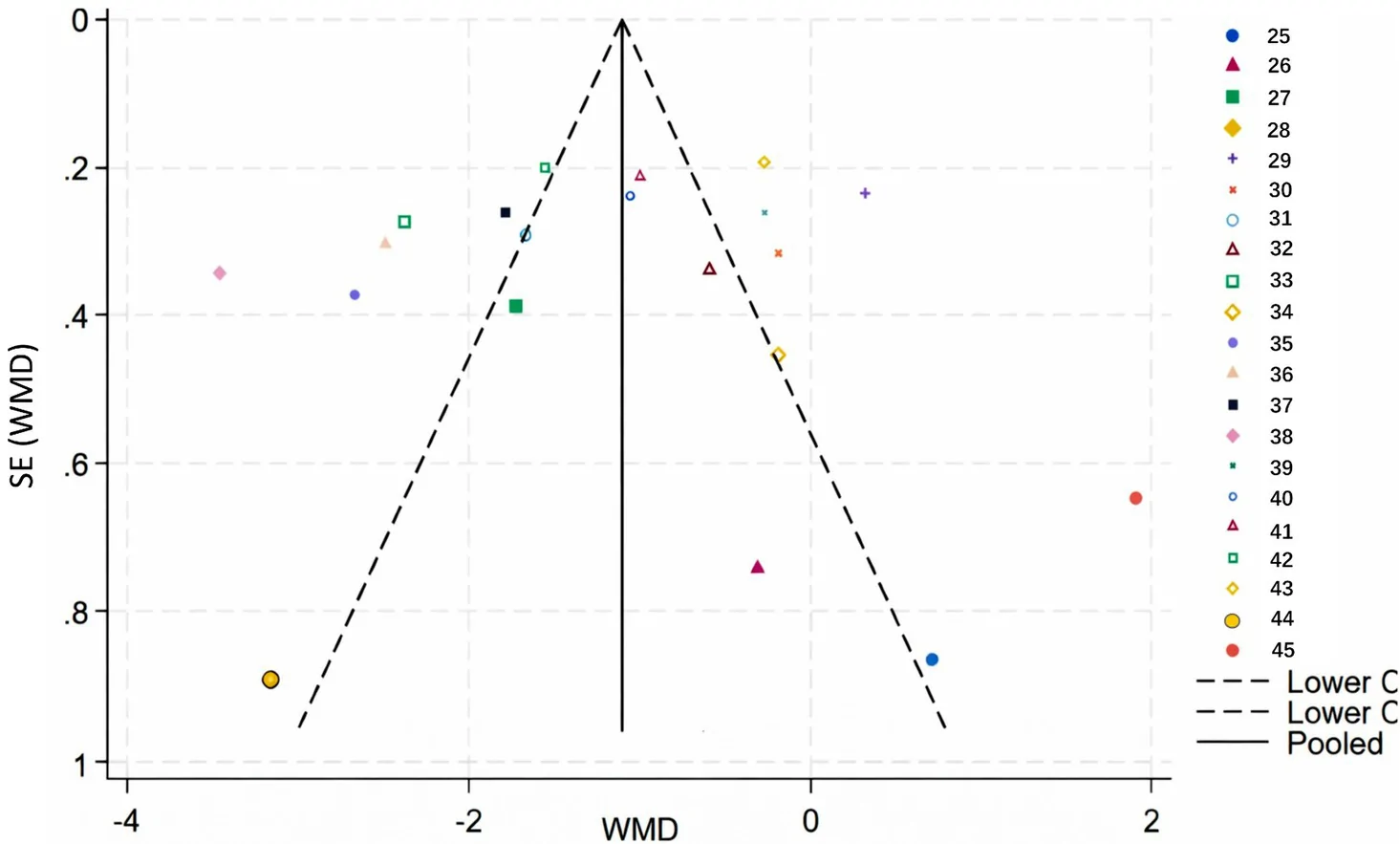
Ferric pyrophosphate (FePP) vs. ferrous sulfate (FeSO_4_) funnel plot.

**Figure 7:**
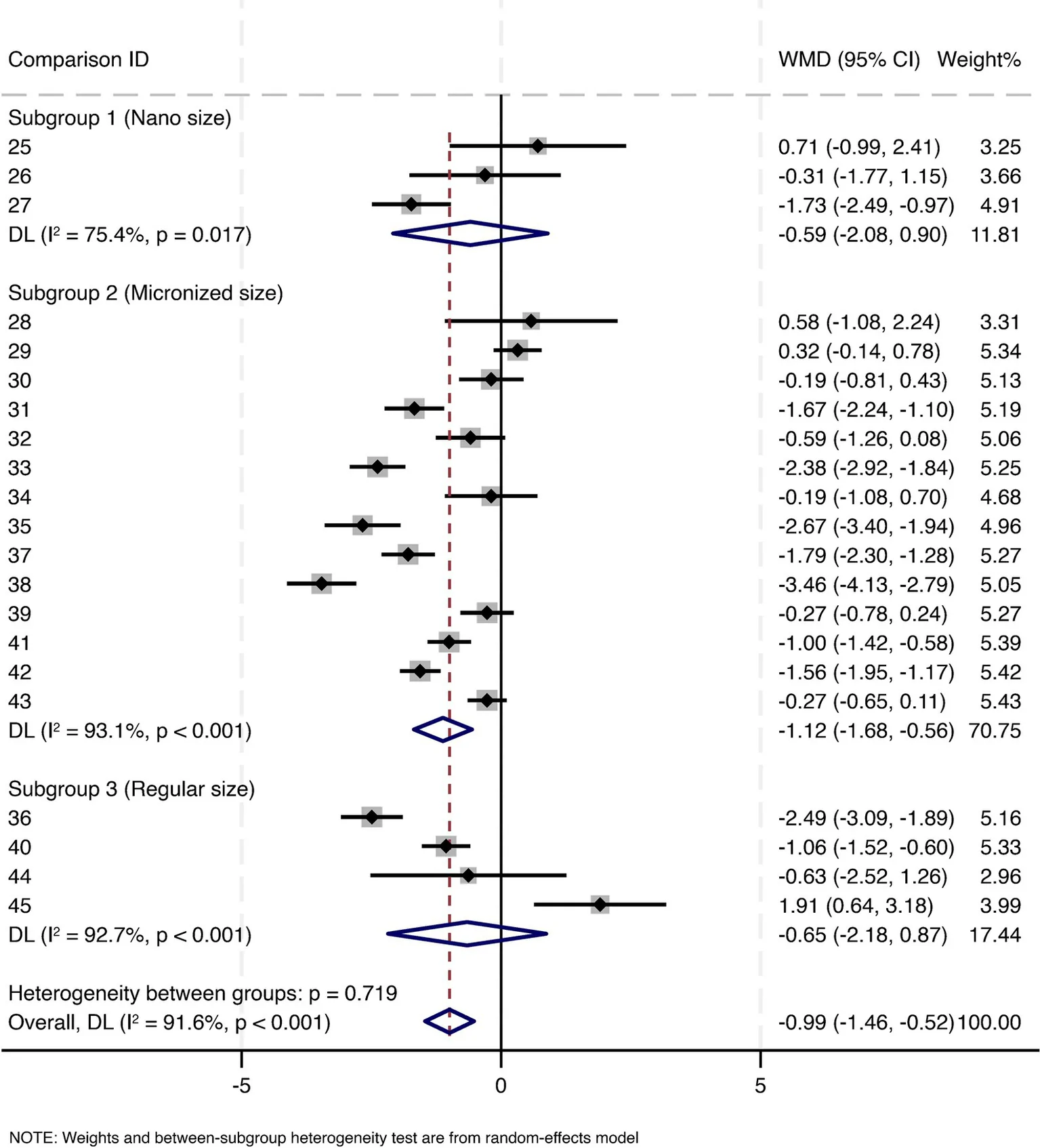
Ferric pyrophosphate (FePP) vs. ferrous sulfate (FeSO_4_) subgroup (particle size) forest plot.

Five studies including 21 comparisons and 360 rats assessed Hb gain (g/dL). Compared to the rats consuming FeSO_4_ fortification, the rats consuming FePP fortification had lower Hb gain (MD = −0.99; 95% CI, −1.46 to −0.52, *p* < 0.001) with high heterogeneity (I^2^ = 92%) (Figure 5). No significant publication bias was found using Begg’s test (*p* = 0.88) and Egger’s test (*p* = 0.72), as displayed in the symmetrical funnel plot (Figure 6). There was no significant subgroup effect on particle size (*p* = 0.719; Figure 7).

#### 3.3.3 NaFeEDTA fortification vs. FeSO_4_ fortification

**Figure 8:**
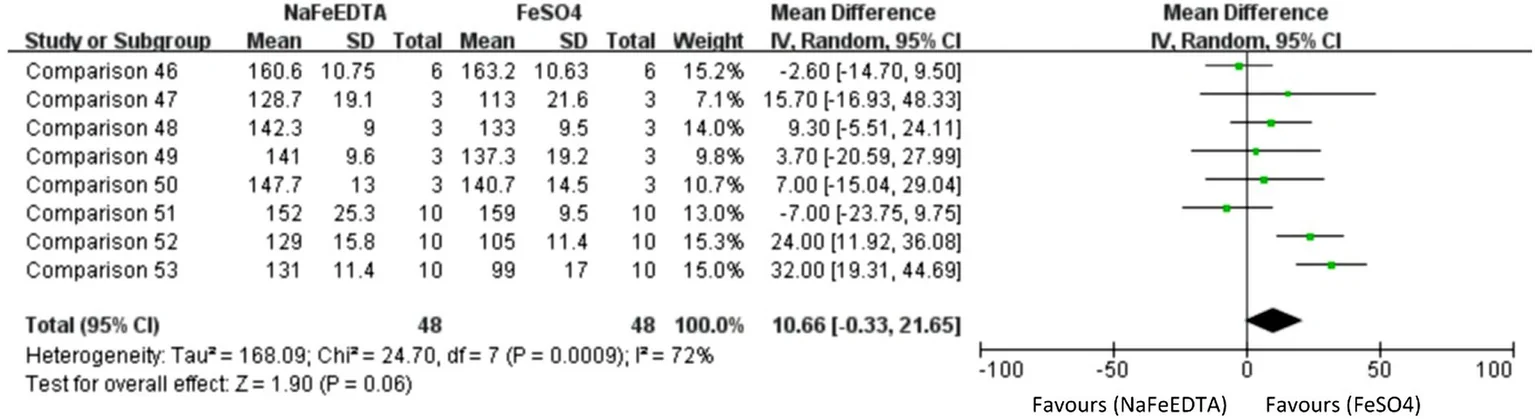
Sodium iron ethylenediaminetetraacetate (NaFeEDTA_)_ vs. ferrous sulfate (FeSO_4_) forest plot.

**Figure 9:**
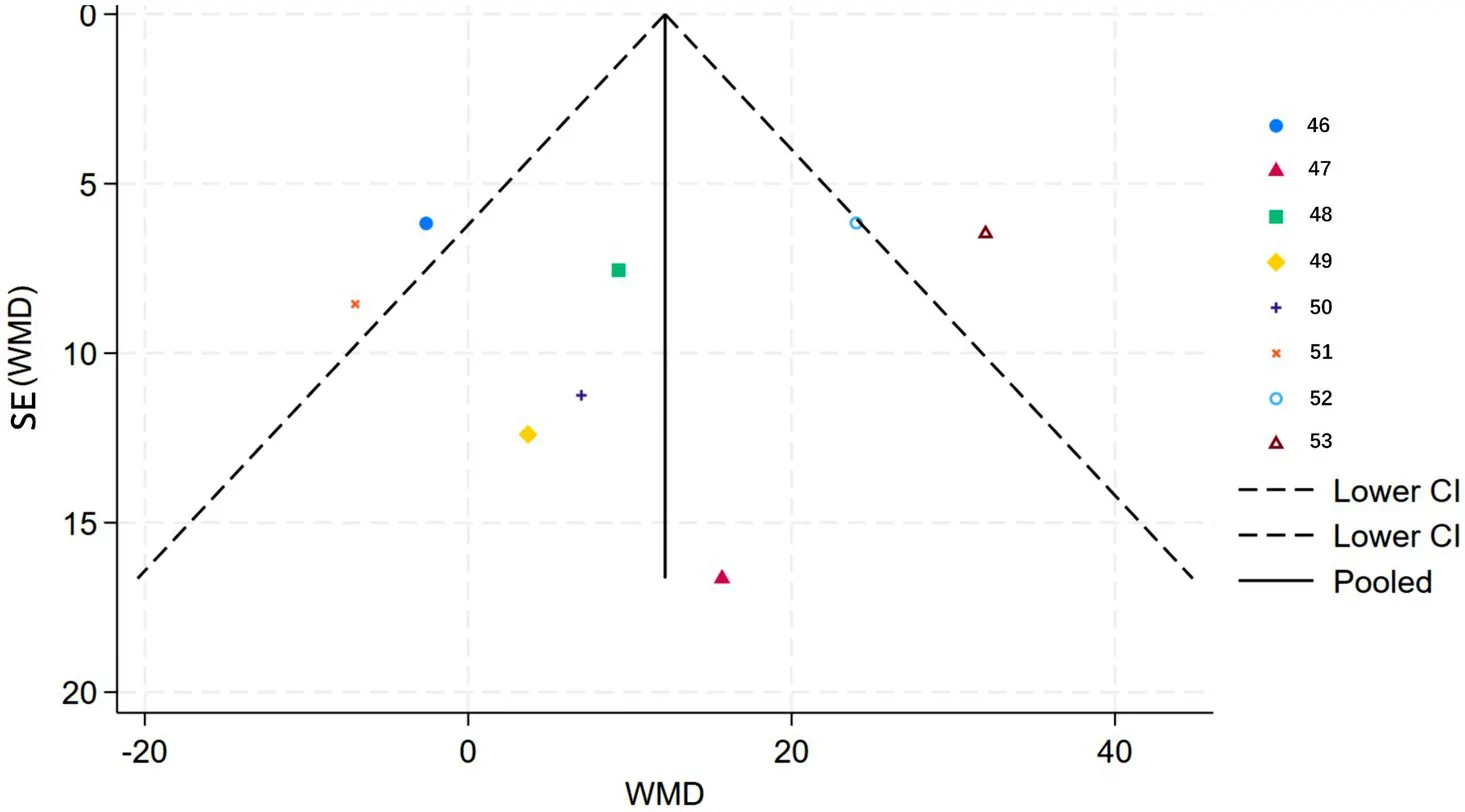
Sodium iron ethylenediaminetetraacetate (NaFeEDTA) vs. ferrous sulfate (FeSO_4_) funnel plot.

Four studies, including eight comparisons and 96 rats, assessed final Hb concentration (g/L). Compared to the rats receiving FeSO_4_ fortification, no significant difference in the overall effect was found in the rats receiving NaFeEDTA fortification (MD = 10.66; 95% CI, −0.33 to 21.65, *p* = 0.06; Figure 8), although there was high heterogeneity (I^2^ = 72%; Figure 3). No significant publication bias was found using Begg’s test (*p* = 0.81) and Egger’s test (*p* = 0.58), as displayed in the symmetrical funnel plot (Figure 9).

#### 3.3.4 Ferrous fumarate fortification vs. FeSO_4_ fortification

**Figure 10:**
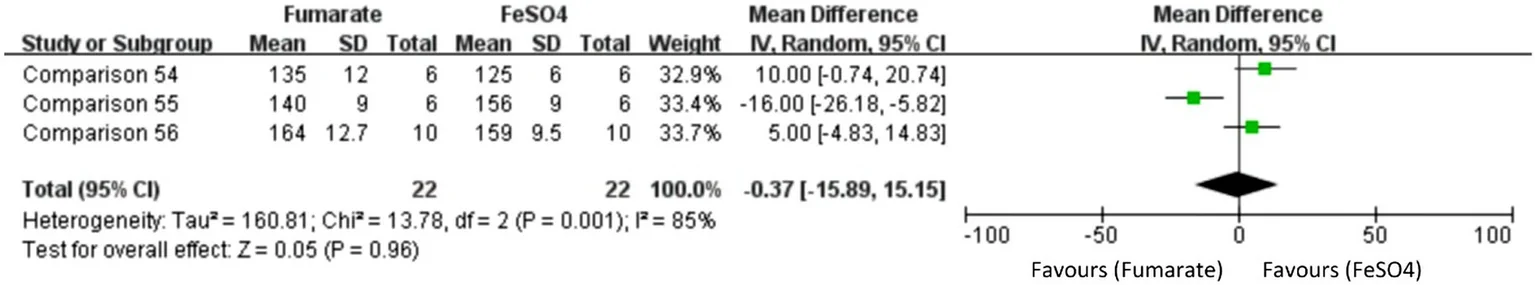
Ferrous fumarate vs. ferrous sulfate (FeSO_4_) forest plot.

**Figure 11:**
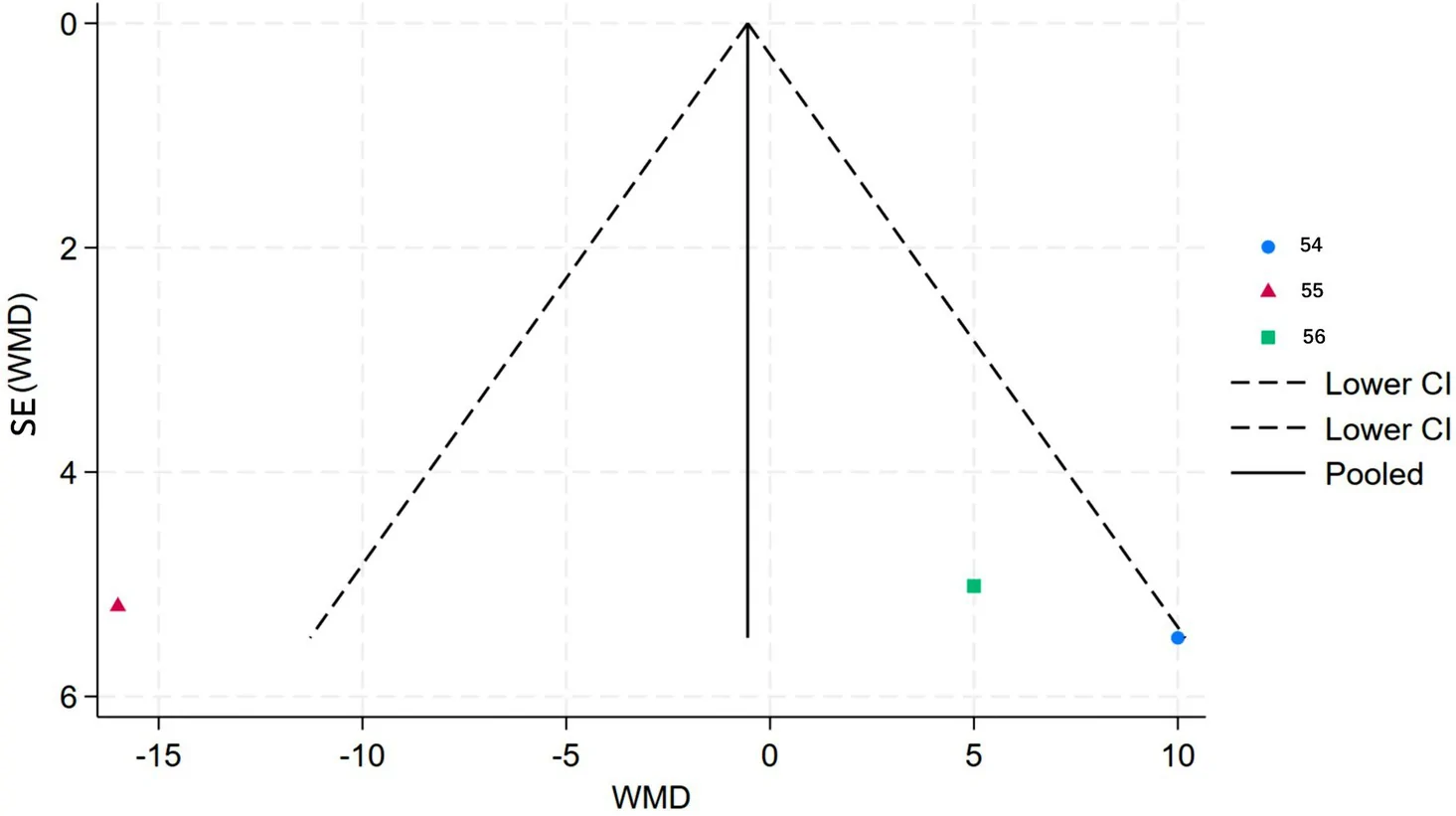
Ferrous fumarate vs. ferrous sulfate (FeSO_4_) funnel plot.

Two studies including three comparisons and 44 rats assessed final Hb concentration (g/L). Compared to the rats receiving FeSO_4_ fortification, no significant difference in the overall effect was found in the rats receiving ferrous fumarate fortification (MD = −0.37; 95% CI, −15.89 to 15.15, *p* = 0.96; Figure 10). A high heterogeneity was found (I^2^ = 85%; Figure 6). No significant publication bias was found using Begg’s test (*p* = 0.60) and Egger’s test (*p* = 0.84), as displayed in the symmetrical funnel plot (Figure 11).

#### 3.3.5 Certainty of evidence

The certainty of NaFeEDTA, ferrous fumarate, and FePP meta-analysis evidence for Hb was evaluated as moderate by the GRADE tool due to moderate or high heterogeneity. However, the certainty of the FePO_4_ evidence was downgraded to low due to high heterogeneity and significant publication bias (Table 6).

**Table 6:** GRADE assessments on Hb of included studies in the meta-analysis.

Iron type	Number of studies	Risk of bias	Inconsistency	Indirectness	Imprecision	Other considerations	Number of animals (intervention group)	Number of animals (control group)	WMD (95% CI)	Certainty
FePO_4_	4	Not serious	Serious	Not serious	Not serious	Dose response gradient; Publication bias strongly suspected	239	185	−1.68 (−2.57, −0.78)	⨁⨁◯◯ Low
FePP	5	Not serious	Serious	Not serious	Not serious	Dose response gradient	180	180	−0.99 (−1.46, −0.52)	⨁⨁⨁◯ Moderate
NaFeEDTA	4	Not serious	Serious	Not serious	Not serious	Dose response gradient	48	48	10.66 (−0.33, 21.65)	⨁⨁⨁◯ Moderate
Ferrous fumarate	2	Not serious	Serious	Not serious	Not serious	Dose response gradient	22	22	−0.37 (−15.89, 15.15)	⨁⨁⨁◯ Moderate

CI, confidence interval; FePO4, ferric orthophosphate; FePP, ferric pyrophosphate; Hb, hemoglobin; NaFeEDTA, sodium iron ethylenediaminetetraacetate; WMD, weighted mean difference. ⨁, one level of certainty.

## 4 Discussion

### 4.1 Characteristics and quality assessments of the included studies

Grain-based diets are usually selected as the food matrix for iron fortification. Their health benefits are partially ascribed to high levels of natural or fortified bioactive compounds. Although the high concentrations of inhibitors in grains such as polyphenols and phytic acid reduce Fe bioavailability, the inhibitors can be largely degraded by some food processing technologies such as extrusion and precooking. Thus, grain-based food products with better nutritional profiles are commercially used not only as a human staple food but also as animal feed (27–29). For example, the results of a Philippines efficacy study showed that wheat flour fortified with ferrous fumarate and vitamin A could improve Hb levels and reduce the prevalence of iron deficiency anemia in school-aged children (30). However, a clinical systematic review, including five studies on the prevention and treatment of iron deficiency anemia using iron-fortified maize flour, did not find evidence that iron-fortified maize flour reduced the risk of iron deficiency anemia or improved Hb concentrations in children and adults (31).

Meanwhile, the positive effect on iron absorption has been demonstrated with the use of micronized and nano-sized fortificants. Nine comparisons showed that nano- or micronized FePO_4_ and FePP were equivalent to FeSO_4_ in terms of Hb levels. Their larger surface area can be more easily hydrolyzed in the gastrointestinal tract. In a double-blind, randomized, placebo-controlled clinical trial conducted in India, the prevalence of iron deficiency anemia in school-aged children was reduced after they consumed extruded rice kernels fortified with micronized FePP for 8 months (32). However, the toxicity of small-sized particles is a concern. For example, nano-sized iron can be readily translocated into body tissues, leading to excessive non-hematological iron uptake, agglomeration, and oxidative stress (33–35). Biomaterials and microencapsulation techniques can address this issue. In a rat study, microencapsulated Fe-polymeric liposome (10 nm FeSO_4_-7H_2_O) had significantly higher Hb. No toxicity was found in this group using histology testing (36).

### 4.2 Pairwise meta-analysis results and certainty of evidence

Due to its excellent iron absorbability, NaFeEDTA, an iron-EDTA chelate, has been recommended by the Joint WHO Expert Committee on Food Additives for government-recommended iron fortification strategies (37). EDTA chelates ferric iron through coordinate covalent bonds, forming an iron-EDTA complex. EDTA can serve as a carrier to protect iron from the ligands of inhibitors in the stomach, thus allowing iron to be better released and absorbed in the duodenum (6, 37, 38). In a rat experiment, NaFeEDTA was significantly more effective than FeSO_4_ in Hb regeneration efficiency, relative iron bioavailability, and average Hb gain (39). Ferrous fumarate is also well absorbed, as it provides iron as Fe^2+^, which is more readily absorbed in the small intestine than Fe^3+^ compounds. In the Caco-2 cell experiment conducted by Wortley et al., encapsulated ferrous fumarate in wheat-cereal vehicle had a higher ferritin response than FeSO_4_ (40). Although FePP and FePO_4_, as two types of water-insoluble iron compounds discussed in this systematic review, did not have strong effects on iron absorption, the nanoparticle techniques including wet chemical methods, dry physical methods, and microbiological processes might improve iron bioavailability. Rohner et al. reported that the specific surface area of regular-sized FePO_4_, larger nanosized FePO_4_ (30.5 nm), and small nanosized FePO_4_ (10.7 nm) were 32.6, 68.6, 194.7 m^2^/g, respectively. The relative bioavailability values in rats were 61, 70, and 96% of FeSO_4_, respectively. No toxicity was found in rats (41).

The significant publication bias found in pairwise FePO_4_ meta-analysis indicated that the studies with positive findings were more likely to be published compared to those with negative findings. As shown in the pairwise meta-analysis plots, high heterogeneity dominated the interstudy variation. Similarly, in a clinical systematic review conducted by Sabitova et al., all I^2^ values in their meta-analysis exceeded 95% (42). In the present systematic review, the moderate and high heterogeneities might be primarily due to study design, including differences in food matrices, particle sizes, doses of interventions, food processing strategies, randomizations, intervention durations, measurement methods and instruments, and statistical covariate adjustments. Thus, moderate confidence was found in the GRADE assessments regarding the overall effects of NaFeEDTA, ferrous fumarate, and FePP meta-analyses. Confidence in the overall effect of FePO_4_ meta-analysis was limited (43).

### 4.3 Limitations and clinical applications

Randomization procedures, blinding methods, and the handling of incomplete outcome data were not clearly described in most studies. These shortcomings might introduce selection, performance, detection, attrition, and publication biases, thereby reducing the reproducibility and internal validity of animal experiments and limiting the applicability of findings (18, 44, 45). In addition, the search strategy for this systematic review was restricted to four databases and English publications, which might have resulted in the omission of relevant studies indexed in other databases or published in other languages.

In animal studies, two models are commonly used: the depletion-repletion model and the preventative-prophylactic model (46, 47). In the present systematic review, due to the restrictive inclusion and exclusion criteria, only 13 studies on iron deficiency anemia were included in the present meta-analysis. Among these included studies, 10 studies used depletion-repletion models, and three studies used prophylactic-preventative models. Other models, such as adenine-induced chronic kidney disease with anemia model, were not included. Although unintended, the predominance of depletion–repletion models improved the comparability of intervention designs across the included studies, thereby strengthening the internal validity of the meta-analysis. However, it might restrict the diversity of the included anemia models, potentially limiting the generalizability of the findings.

Iron bioavailability studies using normal mouse models are rarely conducted because mice absorb iron poorly; however, genetically modified mice are sometimes utilized (48). Therefore, the quantitative assessment of iron bioavailability has been dominated by Sprague–Dawley rat models. Although these animal models provide valuable preclinical evidence under controlled conditions, they may not fully capture the complexity of human iron metabolism, absorption, homeostasis, and physiological responses to iron fortification. Furthermore, experimental conditions are highly standardized and cannot fully account for the genetic, nutritional, environmental, and biological variability observed in human populations. Therefore, caution is warranted when extrapolating these findings to human populations with diverse physiological, nutritional, and clinical characteristics.

It is worth noting that the clinical efficacy of iron fortificants in humans is also being investigated by our team through a network meta-analysis, which is expected to provide further evidence supporting the translation of these preclinical findings into clinical practice. Our team previously conducted a randomized, double-blind clinical trial involving 1,873 Tanzanian children aged 6–53 months. Participants received grain-based food fortified with ferrous fumarate and NaFeEDTA for 5 months. The results showed that iron fortification significantly improved anemia (49). More high-quality preclinical studies and clinical trials are needed to elevate the evidence level of the meta-analysis results, optimize the existing conclusions, examine the safety and efficacy of iron fortification, develop the theories pragmatically, explore new areas of iron bioavailability mechanisms, and develop innovative applications in prevention, diagnosis, and therapy for iron deficiency.

## 5 Conclusion

The meta-analysis results suggest that the overall effects of NaFeEDTA and ferrous fumarate on Hb were equivalent to FeSO_4_. FePP and FePO_4_ were inferior to FeSO_4_. NaFeEDTA and ferrous fumarate were effective in improving Hb concentration.

## Data Availability

The original contributions presented in the study are included in the article/Supplementary material; further inquiries can be directed to the corresponding authors.

## Footnotes

1
https://www.gradepro.org/

## References

[1] Atkinson SH Suchdev PS Bode M Carducci B. Getting back on track to meet global anaemia reduction targets: a lancet Haematology commission. Lancet Haematol. (2025) 12:e717–67. doi: 10.1016/S2352-3026(25)00146-2, 40882649 PMC12774439

[2] Miller DD Berner LA. Is solubility in vitro a reliable predictor of iron bioavailability? Biol Trace Elem Res. (1989) 19:11–24. 2484374 10.1007/BF02925446

[3] Fidler MC Davidsson L Zeder C Walczyk T Hurrell RF. Iron absorption from ferrous fumarate in adult women is influenced by ascorbic acid but not by Na_2_EDTA. Br J Nutr. (2003) 90:1081–5. doi: 10.1079/BJN2003995, 14641967

[4] Ghosh R Arcot J. Fortification of foods with nano-iron: its uptake and potential toxicity: current evidence, controversies, and research gaps. Nutr Rev. (2022) 80:1974–84. doi: 10.1093/nutrit/nuac011, 35263423

[5] Zimmermann MB Hilty FM. Nanocompounds of iron and zinc: their potential in nutrition. Nanoscale. (2011) 3:2390–8. doi: 10.1039/C0NR00858C, 21483965

[6] Zhu L Yeung CK Glahn RP Miller DD. Iron dissociates from the NaFeEDTA complex prior to or during intestinal absorption in rats. J Agric Food Chem. (2006) 54:7929–34. doi: 10.1021/jf0616964 17002472

[7] Churio O Durán E Guzmán-Pino SA Valenzuela C. Use of encapsulation technology to improve the efficiency of an iron oral supplement to prevent anemia in suckling pigs. Animals (Basel). (2018) 9:1. doi: 10.3390/ani9010001, 30577432 PMC6357104

[8] Liyanage C Zlotkin S. Bioavailability of iron from micro-encapsulated iron sprinkle supplement. Food Nutr Bull. (2002) 23:133–7. doi: 10.1177/15648265020233S126, 12362781

[9] Hurrell R. Use of ferrous fumarate to fortify foods for infants and young children. Nutr Rev. (2010) 68:522–30. doi: 10.1111/j.1753-4887.2010.00312.x, 20796217

[10] Ofori KF Antoniello S English MM Aryee ANA. Improving nutrition through biofortification-a systematic review. Front Nutr. (2022) 9:1043655. doi: 10.3389/fnut.2022.1043655, 36570169 PMC9784929

[11] Pantopoulos K. Oral iron supplementation: new formulations, old questions. Haematologica. (2024) 109:2790–801. doi: 10.3324/haematol.2024.284967, 38618666 PMC11367235

[12] Piskin E Cianciosi D Gulec S Tomas M Capanoglu E. Iron absorption: factors, limitations, and improvement methods. ACS Omega. (2022) 7:20441–56. doi: 10.1021/acsomega.2c01833, 35755397 PMC9219084

[13] Pfeiffer CM Looker AC. Laboratory methodologies for indicators of iron status: strengths, limitations, and analytical challenges. Am J Clin Nutr. (2017) 106:1606S–14S. doi: 10.3945/ajcn.117.155887, 29070545 PMC5701713

[14] Rethlefsen ML Page MJ. Prisma 2020 flow diagram. (2020). Available online at: https://www.prisma-statement.org/prisma-2020 (Accessed on March 5, 2026).

[15] Delimont NM Rosenkranz SK Haub MD Lindshield BL. Salivary proline-rich protein may reduce tannin-iron chelation: a systematic narrative review. Nutr Metab. (2017) 14:47. doi: 10.1186/s12986-017-0197-z, 28769992 PMC5525358

[16] Higgins Julian Thomas James. Cochrane Handbook for systematic Reviews of Interventions, (2023). Available online at: https://www.cochrane.org/authors/handbooks-and-manuals/handbook (Accessed on March 5, 2026).

[17] The University of Edinburgh, CAMARADES team. CAMARADES Tools & Resources The University of Edinburgh (2022) Available online at: https://clinical-brain-sciences.ed.ac.uk/camarades/tools-resources (Accessed on March 5, 2026).

[18] Hooijmans CR Rovers MM de Vries RB Leenaars M Ritskes-Hoitinga M Langendam MW. SYRCLE'S risk of bias tool for animal studies. BMC Med Res Methodol. (2014) 14:43. doi: 10.1186/1471-2288-14-43 24667063 PMC4230647

[19] GraphPad Software Limited Liability Company. GraphPad Prism 10 Statistics Guide - Computing the SEM. Available online at: https://www.graphpad.com/guides/prism/latest/statistics/statcomputingsem.htm (Accessed on March 5, 2026)].

[20] Page MJ McKenzie JE Bossuyt PM Boutron I Hoffmann TC Mulrow CD . The PRISMA 2020 statement: an updated guideline for reporting systematic reviews. BMJ. (2021):n71. doi: 10.1136/bmj.n71 33782057 PMC8005924

[21] Swartz MK. The PRISMA statement: a guideline for systematic reviews and meta-analyses. J Pediatr Health Care. (2011) 25:1–2. doi: 10.1016/j.pedhc.2010.09.006, 21147401

[22] Borenstein M Hedges L Rothstein H. Common Mistakes in meta-Analysis and how to avoid them: Fixed-effect vs. Random-Effects, (2023). Available online at: https://www.meta-analysis-workshops.com/download/common-mistakes2.pdf (Accessed on March 5, 2026).

[23] Vesterinen HM Sena ES Egan KJ Hirst TC Churolov L Currie GL . Meta-analysis of data from animal studies: a practical guide. J Neurosci Methods. (2014) 221:92–102. doi: 10.1016/j.jneumeth.2013.09.010, 24099992

[24] Leenaars M Hooijmans CR van Veggel N ter Riet G Leeflang M Hooft L . A step-by-step guide to systematically identify all relevant animal studies (SYRCLE). Lab Anim. (2012) 46:24–31. doi: 10.1258/la.2011.011087 22037056 PMC3265183

[25] Page MJ Moher D Bossuyt PM Boutron I Hoffmann TC Mulrow CD . PRISMA 2020 explanation and elaboration: updated guidance and exemplars for reporting systematic reviews. BMJ. (2020):n160. doi: 10.1136/bmj.n160, 33781993 PMC8005925

[26] Prisma 2020 checklist, (2020). Available online at: https://www.prisma-statement.org/prisma-2020-checklist (Accessed on March 5, 2026).

[27] Johnson PE. Effect of food processing and preparation on mineral utilization. Adv Exp Med Biol. (1991) 289:483–98. 1897405 10.1007/978-1-4899-2626-5_32

[28] Lemmens E De Brier N Spiers KM Ryan C Garrevoet J Falkenberg G . The impact of steeping, germination and hydrothermal processing of wheat (*Triticum aestivum* L.) grains on phytate hydrolysis and the distribution, speciation and bio-accessibility of iron and zinc elements. Food Chem. (2018) 264:367–76. doi: 10.1016/j.foodchem.2018.04.125, 29853389

[29] Paraman I Wagner ME Rizvi SSH. Micronutrient and protein-fortified whole grain puffed rice made by supercritical fluid extrusion. J Agric Food Chem. (2012) 60:11188–94. doi: 10.1021/jf3034804, 23066826

[30] Cabalda AB Tengco LW Solon JAA Sarol JN Rayco-Solon P Solon FS. Efficacy of pandesal baked from wheat flour fortified with iron and vitamin a in improving the iron and anthropometric status of anemic schoolchildren in the Philippines. J Am Coll Nutr. (2009) 28:591–600. doi: 10.1080/07315724.2009.10719791, 20439555

[31] Garcia-Casal MN Peña-Rosas JP De-Regil LM Gwirtz JA Pasricha S-R. Fortification of maize flour with iron for controlling anaemia and iron deficiency in populations. Cochrane Database Syst Rev. (2018) 12:CD010187. doi: 10.1002/14651858.CD010187.pub2, 30577080 PMC6517107

[32] Radhika MS Nair KM Kumar RH Rao MV Ravinder P Reddy CG . Micronized ferric pyrophosphate supplied through extruded rice kernels improves body iron stores in children: a double-blind, randomized, placebo-controlled midday meal feeding trial in Indian schoolchildren. Am J Clin Nutr. (2011) 94:1202–10. doi: 10.3945/ajcn.110.007179, 21940595

[33] Chaudhry Q Scotter M Blackburn J Ross B Boxall A Castle L . Applications and implications of nanotechnologies for the food sector. Food Addit Contam. (2008) 25:241–58. doi: 10.1080/02652030701744538, 18311618

[34] Perfecto A Elgy C Valsami-Jones E Sharp P Hilty F Fairweather-Tait S. Mechanisms of Iron uptake from ferric phosphate nanoparticles in human intestinal Caco-2 cells. Nutrients. (2017) 9:359. doi: 10.3390/nu9040359, 28375175 PMC5409698

[35] Nel A Xia T Mädler L Li N. Toxic potential of materials at the nanolevel. Science. (2006) 311:622–7. doi: 10.1126/science.1114397, 16456071

[36] Cheng J Kenaan A Zhao D Qi D Song J. Photo-polymerizable ferrous sulfate liposomes as vehicles for iron fortification of food. Nanomedicine. (2020) 30:102286. doi: 10.1016/j.nano.2020.102286, 32814175

[37] Bothwell TH MacPhail AP. The potential role of NaFeEDTA as an iron fortificant. Int J Vitam Nutr Res. (2004) 74:421. doi: 10.1024/0300-9831.74.6.421 15743018

[38] Powis G Svingen BA Degraw C. Iron-EDTA stimulated reduction of indicine N-oxide by the hepatic microsomal fraction, isolated hepatocytes, and the intact rat. Biochem Pharmacol. (1982) 31:293–9. 6280724 10.1016/0006-2952(82)90173-3

[39] Whittaker P Vanderveen JE. Effect of EDTA on the bioavailability to rats of fortification iron used in Egyptian balady bread. Br J Nutr. (1990) 63:587–95. 2116895 10.1079/bjn19900145

[40] Wortley G Leusner S Good C Gugger E Glahn R. Iron availability of a fortified processed wheat cereal: a comparison of fourteen iron forms using an in vitro digestion/human colonic adenocarcinoma (CaCo-2) cell model. Br J Nutr. (2005) 93:65–71. doi: 10.1079/BJN20041294, 15705227

[41] Rohner F Ernst FO Arnold M Hilbe M Biebinger R Ehrensperger F . Synthesis, characterization, and bioavailability in rats of ferric phosphate nanoparticles. J Nutr. (2007) 137:614–9. doi: 10.1093/jn/137.3.614, 17311949

[42] Sabitova A McGranahan R Altamore F Jovanovic N Windle E Priebe S. Indicators associated with job morale among physicians and dentists in low-income and middle-income countries: a systematic review and Meta-analysis. JAMA Netw Open. (2020) 3:e1913202. doi: 10.1001/jamanetworkopen.2019.13202, 31922555 PMC6991249

[43] Granholm A Alhazzani W Møller MH. Use of the GRADE approach in systematic reviews and guidelines. Br J Anaesth. (2019) 123:554–9. doi: 10.1016/j.bja.2019.08.015 31558313

[44] de Vries RBM Hooijmans CR Langendam MW van Luijk J Leenaars M Ritskes-Hoitinga M . A protocol format for the preparation, registration and publication of systematic reviews of animal intervention studies. Evidence-Based Preclinical Med. (2015) 2:1–9. doi: 10.1002/ebm2.7

[45] Macleod MR O’Collins T Howells DW Donnan GA. Pooling of animal experimental data reveals influence of study design and publication bias. Stroke. (2004) 35:1203–8. doi: 10.1161/01.str.0000125719.25853.20, 15060322

[46] Forbes AL Arnaud MJ Chichester CO Cook JD Harrison BN Hurrell RF . Comparison of in vitro, animal, and clinical determinations of iron bioavailability: international nutritional Anemia consultative group task force report on iron bioavailability. Am J Clin Nutr. (1989) 49:225–38. 2644802 10.1093/ajcn/49.2.225

[47] Fritz JC Pla GW Harrison BN Clark GA Smith EA. Measurement of the bioavailability of iron, using the rat hemoglobin repletion test. J Assoc Off Anal Chem. (1978) 61:709–14. 649562

[48] Fillebeen C Gkouvatsos K Becker C. Mice are poor heme absorbers and do not require intestinal hmox1 for dietary heme iron assimilation. Haematologica. (2015) 100:e334–7. doi: 10.3324/haematol.2015.126870 25975840 PMC4800685

[49] Delimont NM Vahl CI Kayanda R Msuya W Mulford M Alberghine P . Complementary feeding of sorghum-based and corn-based fortified blended foods results in similar iron, vitamin a, and anthropometric outcomes in the MFFAPP Tanzania efficacy study. Curr Dev Nutr. 3:27. doi: 10.1093/cdn/nzz027, 31143849 PMC6535421

[50] Dickmann RS Strasburg GM Romsos DR Wilson LA Lai GH Huang H. Particle size, surface area, and amorphous content as predictors of solubility and bioavailability for five commercial sources of ferric orthophosphate in ready-to-eat cereal. Nutrients. (2016) 8:129. doi: 10.3390/nu8030129, 26938556 PMC4808859

[51] Lysionek AE Zubillaga MB Salgueiro MJ Caro RA Segal M Shafran N . Bioavailability of petit-Suisse cheese as food vehicle for iron fortification estimated by the prophylactic method. J Nutr Sci Vitaminol. (2002) 48:315–7. doi: 10.3177/jnsv.48.315, 12489824

[52] Srinivasu BY Mitra G Muralidharan M Srivastava D Pinto J Thankachan P . Beneficiary effect of nanosizing ferric pyrophosphate as food fortificant in iron deficiency anemia: evaluation of bioavailability, toxicity and plasma biomarker. RSC Adv. (2015) 5:61678–87. doi: 10.1039/c5ra07724a

[53] Haro-Vicente JF Pérez-Conesa D Rincón F Ros G Martínez-Graciá C Vidal ML. Does ascorbic acid supplementation affect iron bioavailability in rats fed micronized dispersible ferric pyrophosphate fortified fruit juice? Eur J Nutr. (2008) 47:470–8. doi: 10.1007/s00394-008-0750-7, 18953478

[54] Sakaguchi N Rao TP Nakata K Nanbu H Juneja LR. Iron absorption and bioavailability in rats of micronized dispersible ferric pyrophosphate. Int J Vitam Nutr Res. (2004) 74:3–9. doi: 10.1024/0300-9831.74.1.3, 15060895

[55] Wegmüller R Zimmermann MB Moretti D Arnold M Langhans W Hurrell RF. Particle size reduction and encapsulation affect the bioavailability of ferric pyrophosphate in rats. J Nutr. (2004) 134:3301–4. doi: 10.1093/jn/134.12.3301, 15570029

[56] Salgueiro MJ Arnoldi S Kaliski MA Torti H Messeri E Weill R . Stabilized-solubilized ferric pyrophosphate as a new iron source for food fortification. Bioavailability studies by means of the prophylactic-preventive method in rats. Biol Trace Elem Res. (2009) 127:143–7. doi: 10.1007/s12011-008-8229-1, 18802669

[57] Malika S Ullah A Anjum AA Sattar MMK Ali T Manzoor R. Bio-efficacy of iron and zinc fortified wheat flour along with bio-assessment of its hepatic and renal toxic potential. Braz J Biol. (2022) 82:e261695. doi: 10.1590/1519-6984.261695, 35674594

[58] Ologunde MO Morris JB Shepard RL Afolabi AO Oke OL. Bioavailability to rats of iron from fortified grain amaranth. Plant Foods Hum Nutr. (1994) 45:191–201. 8052576 10.1007/BF01094089

[59] Hernández M Sousa V Moreno A Villapando S Lopez-Alarcon M. Iron bioavailability and utilization in rats are lower from lime-treated corn flour than from wheat flour when they are fortified with different sources of iron. J Nutr. (2003) 133:154–9. doi: 10.1093/jn/133.1.154 12514283

