# Supplementary material for: Preclinical perspectives on common iron fortificants and their bioavailability in rat models: a systematic review and meta-analysis
Source: Front Public Health. 2026 Sep 3;14:1902936. doi: 10.3389/fpubh.2026.1902936 (PMC13581748; doi:10.3389/fpubh.2026.1902936)
Supplement: Supplementary file 1 [file Supplementary_file_1.docx]

**Supplementary Materials**

The Supplementary Material for this article can be found online at:

https://www.frontiersin.org/articles/10.3389/fpubh.2026.1902936/full#supplementary-material

**Supplementary Material 1: Flow diagram of steps in the systematic review process**

**Supplementary Material 2: Database search strategy**

(1) Category #1 contained 5 search items: “ferric pyrophosphate”, “ferric orthophosphate”, NaFeEDTA, “ferrous sulfate” and “ferrous fumarate”, to ensure that the testing diet was fortified with one targeted iron additive.

(2) Category #2 contained 18 search items, which ensured that iron status was investigated. They were “iron status”, “iron deficiency”, “iron deficient”, “iron depleted”, “iron depletion”, “iron stores”, anaemia, anemia, anemic, anaemic, microcyt*, hypochrom*, “iron state”, “iron balance”, prophylactic, preventative, repleted, and repletion.

(3) Category #3 included 12 search items: hemoglobin, haemoglobin, hematocrit, haematocrit, ferritin, “total iron binding capacity”, “transferrin saturation”, “serum iron”, “plasma iron”, solub*, “hepatic iron”, “bone marrow iron”, which showed that at least one desired outcome would be reported.

(4) Five search items—forti*, enrich*, food, diet, “micronutrient powder”—in Category #4 indicated that iron additives had to be present in food.

(5) Category #5 had only one search item (trial) to ensure that the included study was not a clinical trial.

In this search strategy, a special syntax was used. The words in the quotation marks could not be separated. The asterisk indicated word variations. All search items under one category were typed into one query search box with “OR” to get a query search result. All query searches were combined with “AND” and “NOT” (#1 query AND #2 query AND #3 query AND #4 query NOT #5 query) in the final search box to obtain the initial articles.
